# User experiences during the transition to calibration-free sensors with remote monitoring while using automated insulin delivery - a qualitative study

**DOI:** 10.3389/fendo.2023.1214975

**Published:** 2023-08-24

**Authors:** Shekhar Sehgal, Martin De Bock, Shirley Jones, Carla Frewen, Benjamin J. Wheeler

**Affiliations:** ^1^ Department of Women’s and Children’s Health, Dunedin School of Medicine, Otago Medical School, University of Otago, Dunedin, New Zealand; ^2^ Department of Pediatrics, University of Otago, Christchurch, New Zealand; ^3^ Pediatric Endocrinology, Health New Zealand (NZ)-Canterbury, Christchurch, New Zealand; ^4^ Pediatric Endocrinology, Health New Zealand (NZ)-Southern, Dunedin, New Zealand

**Keywords:** type 1 diabetes mellitus, continuous glucose monitoring, interview, patient satisfaction, remote monitoring

## Abstract

**Introduction:**

To evaluate the experiences of patients with type 1 diabetes following transition from a calibration-requiring to a calibration-free sensor and remote monitoring in the context of using automated insulin delivery (AID).

**Research design and methods:**

Fifteen participants aged 7–65 years with type 1 diabetes participating in a longitudinal study used a Medtronic® advanced hybrid closed loop (AHCL) device with initially calibration-requiring then calibration-free sensors. Qualitative interviews were conducted ≥20 weeks following use of the calibration-requiring and ≥4 weeks after use of the calibration-free sensors/remote monitoring. Thematic analysis was used to identify key themes and subthemes.

**Results:**

At baseline, mean diabetes duration was 14.5 years ( ± 10.9), mean Hba1c 54.8 mmol/mol ( ± 10.2) (7.2 ± 0.9%) and Time in range 75.4% ( ± 11.6). Participants reported a progressive improvement in digital and lifestyle integration, and device trust following transition to calibration-free sensors with remote monitoring potential. They also reported a reduced need for capillary glucose, increased device satisfaction and trust, and reduced burden of diabetes care. Negative aspects reported included periodic early sensor loss, and for some, impaired integration with mobile devices.

**Conclusion:**

Transitioning to calibration-free sensors with remote monitoring while using AHCL was associated with better user experience, including perceptions of improved quality of life and a reduced burden of diabetes care. Appropriate expectation setting, training, and ongoing support allow for the optimal user experience while using AHCL.

**Clinical trial registration:**

https://www.anzctr.org.au, identifier ACTRN12621000360819

## Highlights

1. What is already known?
  - Automated Insulin delivery devices(AID) can revolutionize type 1 diabetes management and have evolved to incorporate calibration free/reduced sensors, whose impact on user experience in Medtronic devices is unknown.
2. What this study adds?
  - AID with calibration-free CGM was perceived to offer improved system stability, users reported a high degree of trust in the new device, improved quality of life with reduced diabetes care burden.
  - AID with calibration-free CGM reduces overall alarm frequency and burden.
  - Remote monitoring use was more prevalent in children and adolescents than adults, those using felt safer and more aware of glucose levels.
3. How this study might affect research, practice, or policy?
  - This study may assist further research into remote monitoring use in adults as well as enhance the acceptability of AID devices in practice.

## Introduction

Automated insulin delivery (AID) systems have the potential to revolutionize the management and user experience of type 1 diabetes (1). AID consists of a continuous glucose monitor (CGM) and an insulin pump together with an algorithm that provides automated insulin delivery (2). Several studies, using multiple platforms have shown AID to be safe, and improve all aspects of glycaemia (3–5).

In addition to safety and effectiveness, patient experience and usability are key factors to consider. From the trial data, patient reported outcomes including diabetes treatment satisfaction and sleep quality have been shown to improve (6). From the limited qualitative literature, improved perceptions of glycemic control and independence have been found in young adults and their guardians, and trust in an AID device has also been reported as essential (7, 8). However, issues with alarms and sensor calibration notifications are noted to impair user experience (9).

These overall burdens have contributed to a technological drive to introduce calibration-free/reduced CGM systems (1). No study to date has investigated user experience during the transition from AID requiring sensor calibration to a largely calibration-free experience including remote monitoring and automated upload capacity. Both retrospective studies and randomized clinical trials have revealed discontinuation rates of up to 35% in one year in adults using AID devices with calibration requiring sensors (10, 11). The need to calibrate, including the perception that the sensors were nor calibrated correctly were noted to be a factor in 60% of those discontinuing hybrid closed loop and 36% of the sample overall (12). Moreover, having an audible alarm may negatively impact on quality of life, with some young people disconnecting all alarms when socialising (9), and even completely discontinuing automated insulin delivery (12). Recent qualitative work in participants using AID devices have found reduced alarm frequency and sleep disruption following the use of calibration free sensors (13). This has been reinforced by a recent mixed methods study where user and health practitioner satisfaction with the 780G system were high with users experiencing a reduced frequency of alarms (14)

Therefore, in this study, we evaluated real-world user experience, comfort, and level of trust in the device in people with type 1 diabetes who switched from an AHCL system requiring sensor calibration, to one with a calibration-free sensor and additional remote monitoring and automated upload capacity.

## Methods

### Study design

This qualitative study enrolled 15 participants from a longitudinal trial of fifty-four participants designed to determine the effectiveness of an optimization protocol for achieving improved glycemic outcomes while using an AHCL device in a real-world setting. All participants in the longitudinal trial were consented at study outset to participate in a potential qualitative interview and were then selected sequentially using convenience sampling. In this manner, the first 15 sequential consenting participants were selected and interviewed upon completing at least 4 weeks in the second of two study phases.

Recruitment continued until thematic saturation was reached, defined as no new insights in the final two interviews. No participants who had consented subsequently declined to participate/dropped out (100% response/participation rate). The study was approved by the New Zealand Health and Disability ethics committee (ref: 20/STH/214).

### Study participants

Participants in the longitudinal study and this qualitative analysis were aged between 7 and 65 years, had type 1 diabetes mellitus, and prior insulin pump use for ≥6 months. These consisted of 7 adults aged 20-64 years, 1 older person aged 65,5 adolescents/young adults aged 11-20 years and 2 children <11 years. Those with HbA1c >86 mmol/mol(10%) within the 6 months prior to the longitudinal study were excluded, as non-adherence is common in this group, the optimization study required participants with a sustained level of acceptable adherence so that the impact of decision support could be accurately evaluated. Participants were selected using convenience sampling and were approached to organize interview time and setting via email.

### Primary study design

The primary longitudinal study had two phases: phase one utilized research only MiniMed™ 670G 4.0 pump (Medtronic, Northridge California) with an AHCL algorithm and Guardian™ 3 sensors and phase two transitioned to the MiniMed™ 780G AHCL (780G) system (Medtronic) with the calibration-free Guardian™ 4 sensor. Differences between these two devices were the calibration-free Guardian™ 4 sensor, and Bluetooth connectivity. Furthermore, the calibration-requiring sensor required a USB cable to upload sensor glucose information to a cloud-based website (Carelink™) for data analysis and review. By contrast, the system with calibration-free sensor was able to upload data automatically via a connected smartphone using Wi-Fi connectivity several times a day.

### Qualitative interviews

The underlying methodological orientation for this study was thematic analysis. Two qualitative interviews were conducted using topic guides (see **Supplementary Data 1**, **2**). The first interview took place from weeks 20–33 (towards the end of usage of the original devices) and the second interview took place from weeks 37–46 (following at least 4 weeks use of calibration free sensors). Each interview was between 40-70mins duration. Interviews were conducted via Zoom video conferencing app (Zoom LLC, California USA) with the participant based at home and researcher at their workplace with only the participant and interviewer present during the interview. Topic guides were informed by literature review and input from co-investigators. The second interview focused on the transition between the devices using the calibration-requiring to calibration-free sensors and on the participants,’ experiences using the new AHCL device with remote monitoring. All interviews were conducted by S.S. a male endocrinologist and clinical training fellow (MBCHB, FRACP), with experience in qualitative research who was not associated with the patient’s usual clinical care. The interviewer was part of the longitudinal trial research team and participants were aware of the study aims as stated above. This assisted in developing a working relationship and rapport. Participants were aware of the researcher’s interest in Diabetes Technology, as well as the interviewer’s wider research goals. Participants’ interviews were recorded with their consent and transcribed verbatim and subsequently used to generate field notes All participants were invited to review their transcript, however no one requested changes to their interview responses.

### Data analysis

Prior to the first interview, demographic characteristics including age, gender, diabetes duration, pump, socioeconomic status, and CGM use prior to study were collected using questionnaires at the outset of the primary longitudinal study. Interviews were transcribed verbatim by an independent transcriptionist not associated with the study. Both the first and second interviews were analyzed by the lead author(SS) and a nominated secondary author with experience in qualitative research (BW). A coding framework was developed that assisted in capturing themes. Coding occurred line by line to generate initial codes. This involved summarizing distinct ideas within a response utilizing as many codes as needed. Interviews were coded by lead author (SS). A subset of interview transcripts (n=4) was reviewed by a nominated co-author (BW) to consolidate a coding framework for all transcripts. Agreement regarding coding was high between co-authors with discrepancies resolved through consensus. While the protocol allowed for situations where coding did not reach consensus (for discrepant data to be included), for this study this was not actually required. Semantically related codes were used to form themes. NVivo11 (QSR International, Doncaster, Australia) was used to assist in coding and categorization of themes. Each transcript was read by the team of authors who met regularly to discuss thematic insights.

## Results

### Study population

A total of fifteen individuals with type 1 diabetes mellitus were interviewed (Demographics in **Table 1**). **Table 2** summarize study themes common to both interviews with representative quotes.

**Table 1:** Baseline characteristics of interview participants.

Characteristics	Participants
Age	24.9 ± 6.3
Female sex, n (%)	10 (66.7)
Diabetes duration, years	14.5 ± 10.9
Pump use prior to study, n (%)	15 (100)
Duration of pump use, years	7 ± 4.3
Total daily dosage of insulin, U	49.7 ± 21.1
CGM prior to study, n (%)	9 (60)
Proportion of time in auto mode,%	93.7 ± 14.8
HbA1C, mmol/mol (%)	54.8 ± 10.2 (7.2 ± 0.9)
Time in range (TIR%)	75.4% ± 11.6%
NZ Deprivation index 2018^1^	5.4 ± 2.1
Ethnicity	
New Zealand European	11(73.3%)
Māori	1 (6.7%)
Pacific Island	1 (6.7%)
South Asian/Southeast Asian	1 (6.7%)
Latin American	1 (6.7%)
Diabetes complications^2^	1 (6.7%)

Values are mean ± standard deviation, or number of patients (%).

^1^NZDep18 score is a deprivation index based on household address (where the participant lives more than 50% of the time) with one least deprived and ten most deprived.

^2^*Eye complications-moderate retinopathy.

CGM, continuous glucose monitoring; HbA1c, glycosylated hemoglobin.

**Table 2:** Specific feedback from qualitative interview 1 and 2.

Calibration-Interview 1	
Altered schedule (7/15) Takes too long to warm up (7/15)	• ”When I first started it interrupted my sleep because I could only do them at night, so I guess technically I must work my schedule around ……. to take it out and calibrate.” (female, age 24 years) • ”The only downfall is the time it takes for the sensor. For starters you have to charge the sensor then wait for it to calibrate and sometimes that can halt what you had planned.” (female, age 46 years) • ”The time between sensors can be quite long and frustrating.” (female, 28) • ”I come from a perspective of having to test 12 times a day, which is very different to Libre users, so anything like what we have now is a godsend.” (female, age 24 years)
Calibration-Interview 2	
Reduced SMBG requirement (6/15) More user friendly and integrated with life (5/15) More active pursuits possible (2/15)	• “I haven’t had to calibrate at all. I mean I can’t remember the last time it told me to do a test (SMBG) so that’s much easier.” (male, age 12 years) • “I think the difference between the Guardian 3 and the Guardian 4 is that the Guardian 3 felt like it was using like a tool, whereas this feels like it’s working with technology. It’s like you’re working with technology as opposed to using technology.” (male, age 22 years) • “You might be in the middle of doing something fun, and you have to immediately stop and do it. Otherwise, it keeps alarming. So, this new one is amazing because it just lets you live your life.” (female, age 44 years) • “Things like when I go mountain biking or anything like that it’s another thing, I don’t have to have in my pocket so like it’s a small thing but when you’re carrying it around all day with you it actually makes quite a big difference not having to calibrate.” (male, age 22 years)
Alarms interview 1	
Alarm fatigue (3/15) Alarms disturbing (3/15) Alarms causing sleep disturbance (2/15)	• ”We do get annoyed about the number of beeps.” (parent of female aged 9 years) • ”Sleep is a classic example for sure. I always try and minimize the alarms as much as I can because it does get disruptive or distracting. I guess because I’ve been a person with diabetes so long that it’s like I know that the closed loop system is going to handle this high. Not that I don’t want to know, but I don’t know if I need to know as much if I’m 13.” (female aged 28)
Alarms interview 2	
Reduced Alarms (4/15) Alarms annoying and ignored (2/15)	• ”I found with the calibration that it kept waking me up in the night to calibrate it and do all sorts of different things, so it’s reduced the number of alarms that go off by a substantial amount.” (male, age 22 years) • ”We have put on alert before Hi which I think has been good, but I have a feeling that *name* might ignore it a bit at school.” (mother of female aged 12 years)
Sensors interview 1	
Shortened Sensor Duration(7/15)	• “Having a longer duration sensor such as 10 or 14 days would be ideal.” (male, age 36) • “Had one point where it was like three or four days in and it just decided to stop working and it was like well, I’m I don’t carry spare sensors with me.” (male, aged 22)
Sensors interview 2	
Shortened Sensor Duration persists (9/15)	• “I feel like I’ve had a lot more issues with sensors themselves. I’ve been finding that the sensors aren’t always lasting the seven days.” (Female, age 44) “The previous one I was on (isCGM) lasted for 10 days, similar for Dexcom. Sometimes the sensor life is three or four days, but others last for 6 days; the previous Guardian 3 sensor transmitters lasted longer.” (Male, age 17 years)
Trust in Device interview 1	
Complete trust in the calibration requiring Device (10/15) Intermediate trust-trusting at times (3/15)	• ”I have a lot of confidence in the device. I like how it has a really good user interface. I trust it to keep an eye on things. I’m confident that it will inform me if something is a little bit out of whack.” (Female, age 24 years) • ”Yeah, I trust it … both, completely. I’m pretty comfortable with my other automated devices as well.” (Female, age 44 years) • ”The times I don’t trust it are the times that I know that it can’t cope, which is like if I’m going hunting or tramping and I’m walking up a hill with a backpack on so that’s when I’ve got to keep a bit of an eye on it, but during regular living (normal activities) I have complete trust because it’s perfectly fine and can handle it easily.” (Male, age 22 years) • ”I find that with the AHCL, I hate not having control, I find with my level of knowledge and experience with diabetes I should be able to fine tune things to a better extent “(female, age 28 years)
Trust in Device interview 2	
Trust in calibration free sensor (15/15)	• ”I’ve been actually doing yoga recently with my pump on because I trust it.” (Female, age 24 years) • ”I really do trust it. I definitely sleep better now because before it was a matter of getting your blood sugar perfect and stable before you go to sleep.” (Female, age 38 years)

AHCL, advanced hybrid closed loop.

### Setting the scene - goals of entry: reasons for entering the study

The main reasons given for entering the primary longitudinal study were the chance to improve glucose levels (12/15 respondents), reduced finger prick frequency (4/15), improved quality of life (3/15) and helping science by assisting in research. Some participants had specific concerns regarding hypoglycemia frequency (quotes 1-3).

• ”I guess I worried about the highs and what they would mean for me long term but the lows I was actually scared of because those have been really where my problem area is” (female, age 24 years).

• ”I think that the calibrations are a necessary evil. I hate having to prick my finger. But this is nothing compared to the amount that I was doing at the beginning of my time with diabetes” (female, age 24 years).

• ”I have eye issues and with more control, the more control I get with my diabetes, the better the complications (female, age 34).

### Calibration experience: a pathway to increased freedom

As context, all participants using AHCL had prior experience with insulin pumps, so none found the education process too challenging. Compared with other insulin pumps (spanning multiple manufacturers), many felt that learning to use the AHCL system was straightforward, and they felt well supported by the study team overall (**Table 2**).

Participants reported that the calibration-requiring sensor required multiple calibrations that would often result in several hours without a sensor glucose reading, triggering alarms overnight and interrupting sleep (**Table 2**). Most respondents found this to be a negative experience, with 7/15 stating that the sensor took too long to warm up, and a further 7/15 reporting that this process altered their plans for the day. However, participants were willing to acquiesce to finger prick testing if it resulted in access to a closed-loop device (described as a “necessary evil” by one respondent – quote 2).

### Increased freedom

The transition to calibration-free sensors led to an increased sense of freedom. Six participants commented positively about the reduction in SMBG requirement. Two younger participants found that they could undertake more active pursuits such as mountain biking without having to take their finger pricker/meter (**Table 2**). All participants found having to do fewer SMBG measurements more convenient (**Table 2**). As a result, some perceived the device as more user friendly and more integrated with their lives, increasing perception of device satisfaction.

### Alarm frequency and alarm fatigue

Overall, there was a mixed response to the alarms and alerts during both study phases. Nine participants found the alarms to be beneficial to various degrees; many stating that alarms were rare and did not significantly interrupt their daily lives. Three participants had varying degrees of alarm fatigue, another was reluctant to add further alarms, and another two had to actively adapt alarms to ensure that these did not interfere with sleep (**Table 2**). Many adolescents found the alarms annoying (**Table 2**).

Following the change to calibration-free sensors, a perceived reduction in alarm frequency was observed. Some participants still reported mixed experiences with alarms and alerts. Four participants (4/15) mentioned significantly fewer alarms with the 780G, this specifically included reduced calibration alarms (**Table 2**). Two participants (2/15) mentioned that alarms were annoying, and one parent (1/15) mentioned that their daughter may still ignore alarms at school (**Table 2**).

### Trust in the device

Upon re-interview, all participants stated that they trusted the calibration-free sensors to provide accurate glucose levels. As a result, some felt confident enough to attempt new activities such as yoga and many felt confident enough to leave their finger prick devices for calibration at home (**Table 2**).

Most participants stated that they trusted the calibration free sensor to a high degree or completely (10/15 respondents). Those who trusted the sensor stated that familiarity with the device over time allowed trust to be gained (**Table 2**) and trust in the manufacturers of the sensor was also mentioned as important (**Table 2**). Many participants stated that confidence in the device allowed them to gain healthier glucose levels (**Table 2**). Of the five remaining participants, four mostly trusted the calibration-free sensors. In one case there was a slight lack of perceived trust in the sensor accuracy when sensor glucose was low, but this did not impact on the overall dimension of trust in the calibration-free sensor. The majority of those with an intermediate degree of trust (3/15) trusted the calibration-free sensors at night, when there were fewer glycemic variables compared with daytime (**Table 2**); one participant found it difficult to rely on the sensor glucose values during vigorous outdoor exercise (**Table 2**).

### Sensor duration

Many participants (7/15) reported that the sensor duration was shorter than the reported seven days, often spanning between 3 - 6 days (**Table 2**). Transition to a calibration-free sensor did not appear to change this, indeed shortened sensor duration appeared to persist. This was perceived to be the primary shortcoming of the sensors (**Table 2**).

### Remote monitoring experience

An enhanced feature of the use of calibration-free system was the ability for parents and other interested parties (care partners) to view sensor glucose data in real time (follow) using the Care Partner app. We found, during the follow up interviews, that 5/5 (children and adolescents) activated this feature, enabling their caregivers to follow their glucose data. Those participants that did use the follow feature had a positive response to it and found that it led to increased safety and enhanced shared understanding of glucose patterns (quote 4 and 5).

Only 2/10 adult participants and their care partners (spouse) activated the follow function (quote 6); some found the feature was not essential and others still felt a sense of threatened independence with external overview of their glucose levels (quotes 7-9).

• ”I understand the goal well the advantage would be to have her with a master and myself with a follower, so I haven’t been able to use the following app yet.” (Parent of female age 9)

• ”It also gives me a better understanding of what Dad wants and what I want and then we can kind of work it out between the two.” (Male, aged 17)

• ”He’s got those notifications that come up on his phone straight away when something goes wrong. And that actually works for both of us really well.” (Female, age 34)

• ”I think if I was to use it, it would be quite gimmicky.” (Female, age 19).

• ”No, I haven’t used the following app at all.” (Male, aged 36)

### Design, menu, and phone/device connectivity

A further new feature of the calibration-free system was the ability to upload data wirelessly via a connected smartphone. The overall user experience of this was positive, especially in older participants (quote 10), some connectivity issues remained and included problems with phone app activation and it is taking too long to upload data (15 minutes vs. 1–2 minutes) (quote 11). One user had to default using her laptop to download, a method which offered no significant advantage over the prior calibration requiring system (quote 12).

• ”It just sends the information up for me … which is fantastic not having to remember always to have to do that and what’s nice is I can just tap on my app, and I can just look, it gives me all the information in one page.” (Female age 65)

• ”Its actual connectivity is really bad, so it takes upwards of 15 minutes to actually connect with the pump and then it does not stay connected.” (Female, age 28 years)

• ”The only thing that was tricky was my phone for some reason couldn’t download the app or use the app, but we got a dongle instead and that’s how I’ve been uploading.” (Female, age 38)

Two adult participants (2/10) mentioned that it would be useful to be able to read their glucose levels on a smartwatch.

• I wish that the app went to my watch so that I could also view the glucose levels on my smartwatch (male, age 36).

### Overall quality of life and burden of care

Nine participants (9/15) reported improvement in their quality of life using the new AHCLsystem. Most attributed this to increased freedom or reduced burden of daily diabetes self-management tasks (e.g., calibration). They cited the ability to trust that the closed loop was working in the background, as the calibration-free CGM was providing accurate sensor glucose information without the need to calibrate. Parents (4/5 parents interviewed) noted that the combination of having a calibration-free device and remote data monitoring allowed them to spend less time focusing on diabetes and more time focusing on other aspects of life (quotes 14 -17).

• ”it gives you much more freedom, much more freedom of mind, because you’re not having to finger or check your pump. It gives you a feeling that you can live your life better, or better quality of life. Because it’s, you know, that it’s more in control.” (Female, aged 60)

• ”I feel a lot more freedom with my diabetes than I ever have before.” (Female, aged 34)

• ”Not that you forget that she has diabetes, but you know that you don’t have to be fully focusing on it while you are doing things.” (Mother of aged 8)

• ”The autocorrections are working really well … I might have like a piece of chocolate or something like that….it corrects for it.” (Male, aged 18)

## Discussion

This qualitative study summarizes the views of an experienced cohort of AID users who transitioned from an AID system with a sensor requiring calibration to one that didn’t need calibration, and could auto-upload data with remote monitoring. The majority of participants reported an improved user experience with a high degree of trust in the device together with improved quality of life and a reduced burden of diabetes care. Alarm fatigue reduced progressively throughout the study, especially when calibration-free sensors were introduced. Many participants found that the requirement to finger prick twice a day in the first phase of the study was removed to a substantial extent by transition to a calibration-free sensor, and that in general led to perceived quality of life improving.

We anticipated an incremental improvement in device satisfaction, sleep, and device trust with a calibration-free AID system. This was confirmed by data from the second interview where most participants reported a much-reduced need for finger prick glucose levels and much faster calibration when required, which resulted in less down time and reduced burden of care. This was associated with improvements in device trust and participant quality of life. Many younger users felt that these features also resulted in better integration of the device with their lifestyle, enhancing their sense of freedom and reducing the overall burden of diabetes. This was in contrast with findings from a previous single-center study showing youth discontinuation of the predecessor AID system (Medtronic® 670G) that used the calibration-requiring sensor, due to frequent calibration requirements and time burden (12).

Many young people with diabetes prefer not to be involved in visible diabetes self-care tasks for a variety of reasons, including peer pressure, risk taking and body image (9). Our data are consistent with the findings of another qualitative study showing that use of a closed-loop system with calibration free CGM enabled younger participants to feel more in control of and less overwhelmed by their diabetes (7). In addition, our findings extend these observations to adults. Auto uploading is another method where a potentially visible diabetes self-care task is automated. This is an important feature of the studied device and was received favorably.

The ability to share data remotely via the Care Partner app (follow) was a novel feature introduced by the upgraded AID system (15). Previous work in youth with real time continuous glucose monitoring (rt-CGM) found that those who had at least one follower had improved glycemic control and device satisfaction (16). An early study in children utilizing CGM with remote monitoring revealed improved parental fear of hypoglycemia and quality of life (17). A further study in adolescents has found that those using CGM with remote monitoring had modestly improved glycemic control compared to users that did not use remote monitoring (18). This has been further reinforced by a retrospective study in 15,000 youth where those with a follower/care partner had improved glycemic control and device utilisation (15). Interestingly, we further found a reduced uptake overall of Care Partner/follow, especially in adult participants, with a number of contributing factors in these participants including perceived lack of technical competence and a desire to preserve independence and maintain personal boundaries. There is limited literature in this field in adults, and could potentially represent opportunities for future work to fully understand this.

Along with the positive features of the new sensors, users also reported some negative aspects. One of these was alarm fatigue, which is a common reason for discontinuing use of AID and RT-CGM (19–21). Many users felt restricted by the device alerts for both hyperglycemia and hypoglycemia and felt that the former impaired sleep. Many participants stated they would prefer to have fewer alerts – and reduced calibration alerts helped with this. Previous studies have found that the presence of fewer alarms, with a higher severity threshold could reduce alarm fatigue and enhance patient safety. This in turn requires close coordination between persons with diabetes and their clinicians (22, 23). The need for a personalized approach to alert/alarm settings form the cornerstone of recently published adult and pediatric clinical guidelines that address continuous glucose monitoring (24, 25)

Another issue raised by participants was a perceived reduced duration of sensor use, that was reported to range from 3 to 6 days for both sensor types, leading to some user frustration Sensor life and reliability remains an area for improvement in many systems with up to 32% of CGM and 24% of intermittent scanned continuous glucose monitors(isCGM) ending prematurely (26) In contrast earlier work on the retention and reliability of rt-CGM sensors has revealed rates of retention between 87-95.7% over 10-14 days depending on sensor type used (27–30). In the case of Dexcom sensors, most cases of reduced sensor survival were due to early sensor shut off (29). Connectivity with newer phones and associated operating systems is a challenge for industry to keep up with and could further contribute to lower device satisfaction. Digital integration is an important consideration as previous studies have shown that AID users expressed a desire to see glucose data on their phones, which form an indispensable part of their daily lives (9).

### Strengths and limitations

The strengths of the study included the use of two interviews to capture the opinions of a cohort of pump users transitioning to AHCL who, for the first time, also transitioned between a sensor requiring calibration and one that did not. In contrast, previous qualitative work in AID devices have compared predictive low-glucose management (PLGM) to AID (7, 31), and previous work in real-time (RT-CGM) has focused on comparisons with capillary glucose (32, 33).

In terms of limitations, the primary study inclusion criteria, and use of convenience sampling, together with the primarily European population meant that the cohort may not be representative of the wider type 1 diabetes population. The study also excluded those with very poor control(HbA1C>86)(10%), as a result the study may not be generalizable to that group. Using two interviews also meant that there could be a crossover effect in terms of the themes of device satisfaction and trust, and the second interview occurred in what could be referred to as ‘the honeymoon period’ with the second device.

## Conclusion

Calibration-free AID is associated with an improved user experience through a reduced burden of care and high degree of trust. Areas for improvement include sensor lifespan, connectivity, and digital integration.

## Data availability statement

The raw data supporting the conclusions of this article will be made available by the authors, without undue reservation.

## Ethics statement

The study was approved by the New Zealand Health and Disability ethics committee (ref: 20/STH/214). Informed consent was obtained from all subjects and/or their legal guardian(s) for participants below 16 yrs of age.

## Author contributions

SS was responsible for patient interview, development of the coding frame and preparation of the first draft of the manuscript. BW and MD assisted in developing the coding frame, reviewing the transcripts and editing the manuscript prior to submission. SJ and CF assisted in patient recruitment. All authors approved the manuscript for submission.
