# Supplementary material for: User experiences during the transition to calibration-free sensors with remote monitoring while using automated insulin delivery - a qualitative study
Source: Front Endocrinol (Lausanne). 2023 Aug 24;14:1214975. doi: 10.3389/fendo.2023.1214975 (PMC10484395; doi:10.3389/fendo.2023.1214975)
Supplement: Supplementary file 1 [file Table_1.docx]

**Supplementary File ONE**

Details of topic guide questions for interview 1

| **1.** Tell me what about this study interested you?   - Is there is a problem you thought the tech might make better/easier/go away? - Hypoglycaemia - Hyperglycaemia - Less finger prick blood testing - Did you have a goal you were trying to achieve? - Overall reasons for using an AHCL device? Examples: - Manage glucose levels better when playing sports - Improved HbA1c |
| --- |
| **2.** How did you expect your life to be different when that problem was solved/goal was achieved? |
| **3.** Discuss your training experience. How easy was the AHCL device to use and learn? Is there anything that you would recommend for training new people? |
| **4.** Was your experience using AHCL as you expected?   - If they expected improved glycaemic control, did that happen? - What new habits were formed because of AHCL? - What about the system helped? (e.g., did something new become a habit? how did it become a habit?) - If they expected improved quality of life, did that happen? (or prompt about quality of life?) - What about the system helped? - Thinking about the recommendations for using AHCL at the start of the study, what did you try that didn't become a habit? What were the reasons for it not becoming a habit? - What was recommended that you didn't try at all? What were your reasons? |
| **5.** Were there situations when your experience using the AHCL device was better or worse?   - e.g., daytime vs night-time, in public vs private spaces, or when their sleep/mood/stress/fear of hypos was better/worse, or with/without support from partner/whanau/friend |
| **6.** What experiences with using AHCL were unexpected? (e.g., barriers/facilitators to improved glycaemic control/quality of life)   - Follow-up: what problem-solving did you need to do in this situation? - If not already mentioned, ask directly: - What was your glucose target? Why this one, and if on the lower - did the lower target glucose range impact them, how? (Was it low enough?) - If not already mentioned, ask directly: - Tell us about your experiences with auto-correction boluses? Better or worse than expected? - How did AHCL cope with different meal types? (i.e., high fat/high carbohydrate/high protein, etc) |
| **7.** Overall - How does AHCL compare to your previous treatment? Specifically:   - Changes to glucose level monitoring habits - Changes to insulin treatment habits - Changes to overall habits related to diabetes examples being exercise, sleep, worry, daily activities, work, social/family interactions - Helped/hindered ability to maintain glucose levels in target range - Made managing diabetes easier/harder - Does it need more or less clinical support from your diabetes team? Why? |
| **8.** Would you recommend AHCL to other people with type 1 diabetes?   - Why/why not - In your opinion who could benefit the most from AHCL? Why? - Who might not benefit from AHCL? Why? |
| **9.** Do you trust the AHCL device? Please explain.   - What makes you trust AHCL? - Why don’t you trust AHCL? - Do you trust AHCL only in specific circumstances |
| **10.** Is there anything else you’d like to share with me today? |
