# Supplementary material for: User experiences during the transition to calibration-free sensors with remote monitoring while using automated insulin delivery - a qualitative study
Source: Front Endocrinol (Lausanne). 2023 Aug 24;14:1214975. doi: 10.3389/fendo.2023.1214975 (PMC10484395; doi:10.3389/fendo.2023.1214975)
Supplement: Supplementary file 2 [file Table_2.docx]

**Supplementary file 2**

**Repeat AHCL interview Topic Guide**

**1. Overall Experience**

You’ve now been using the Guardian 4 transmitter and sensor combination for 4 weeks , How has your experience changed since transitioning from Guardian 3?

a) Has the change been for better or worse?

**2. Uploading**

What has your experience of uploading been like when you upload your new device(s) compared to your old one?

**3. Calibration and Calibration related QOL**

How does the process of calibration differ between the two sensors?

what aspects of daily life have been made easier? What have been made more difficult?

Have using the new sensor/transmitter combination improved your quality of life?

**4. Follow and Care Partner**

How have the both of you found using care partner ?

Has it changed your relationship with your care partner? If so what aspects?

How has the information provided by care partner app changed the way you as a care team understand your partner’s diabetes ?

**5. Trust in the AHCL device**

To what extent do you trust your AHCL device?

Has that changed in the last 4 weeks ?

If there has been change what aspect(s) of the device have driven this change?
